# The Influence of Threatened Miscarriage on Pregnancy Outcomes: A Retrospective Cohort Study in a Nigerian Tertiary Hospital

**DOI:** 10.7759/cureus.31734

**Published:** 2022-11-21

**Authors:** Ubong B Akpan, Chinyere J Akpanika, Udeme Asibong, Kazeem Arogundade, Adaolisa E Nwagbata, Saturday Etuk

**Affiliations:** 1 Department of Obstetrics and Gynaecology, University of Calabar Teaching Hospital, Calabar, NGA; 2 Department of Family Medicine, University of Calabar Teaching Hospital, Calabar, NGA; 3 Department of Public Health, Bruyere Research Institute, Ottawa, CAN

**Keywords:** bed rest, placenta previa, caesarean section, vaginal bleeding in pregnancy, threatened miscarriage, spontaneous miscarriage

## Abstract

Background: Pregnancies complicated by threatened miscarriage (TM) may be associated with adverse pregnancy outcomes. The objective of this study was to compare the differences in pregnancy outcomes between the women who experienced TM and asymptomatic controls.

Methods: This was a 10-year retrospective review. Case records of 117 women who were managed for TM from January 1, 2010, to December 31, 2019, were retrieved and studied. The control group was developed from an equal number of asymptomatic clients matched for age, parity, and BMI who were receiving antenatal care (ANC) during the same period. Data on demography, clinical and ultrasound findings, treatment, and pregnancy outcomes were retrieved and analyzed.

Results: Spontaneous abortion rate of 13.7% was recorded among the study group compared with 3.4% in the control (P-value [p] = 0.005, odds ratio [OR]: 4.475; 95% confidence interval [CI]: 1.445 - 13.827). Women with TM had higher odds for placenta previa (p = 0.049, OR: 4.77, 95% CI: 2.19 - 23.04), premature rupture of membranes (PROM) (p = 0.028, OR: 1.918, 95% CI: 1.419 - 2.592), postpartum hemorrhage (PPH) (p = 0.001, OR: 2.66, 95% CI: 20.8 - 8.94), and preterm birth (OR: 2.5, 95% CI: 1.75 - 3.65). They were also more likely to undergo cesarean section (p = 0.020, OR: 1.70, 95% CI: 1.053 - 2.964). There was no statistically significant difference in their infants’ mean birth weight (3.113 ± 0.585kg for the TM group and 3.285± 0.536kg for the control, P=0.074). Other maternal and perinatal complications were similar. Admission for bed rest significantly improved fetal survival. Women who were not admitted for bed rest had higher odds of pregnancy loss (OR: 3.443, 95% CI: 1.701-7.99). Other treatment plans did not significantly contribute to a positive outcome.

Conclusion: Threatened miscarriage is a significant threat to fetal survival and may increase the risk for operative delivery. Bed rest improves the live birth rate.

## Introduction

Vaginal bleeding in the first half of pregnancy is a common problem encountered in obstetrics practice and is reported to occur in about one-fifth of all pregnancies [1,2]. It is one of the visible warning signs in pregnancy but oftentimes, most of such pregnancies progress to live birth with good maternal and fetal outcomes [3,4]. Studies have shown that this pregnancy event is among the most common indication for admission for in-patient care during pregnancy [5,6]. Threatened miscarriage is characterized by vaginal bleeding, often painless, associated with a closed cervix and ultrasonographic evidence of fetal cardiac activity occurring before 20 weeks gestational age [7].

Meta-analysis indicates that vaginal bleeding in pregnancy is associated with a two-fold increased risk of complications such as pregnancy-induced hypertension, preeclampsia, preterm rupture of membranes, spontaneous abortion, repeated and late pregnancy bleeding, placenta previa, retained placenta and postpartum haemorrhage. About half of the bleeding episodes during pregnancy have unknown causes and it may also be difficult to predict which of such women would develop complications in the course of the gestation [8,9], although these complications are more in women with underlying medical conditions such as maternal obesity and chronic hypertension.

Threatened miscarriage is a known cause of anxiety among pregnant women who experience it and their caregivers. It also contributes to an increased financial burden in caring for pregnancies. Vaginal bleeding is a common reason for a referral from peripheral centres to the Maternal Fetal Medicine unit of our facility for specialist consultation in the first half of the pregnancies. Several interventions may be attempted to improve pregnancy outcomes in these patients including bed rest and medication such as progesterone; however, the extent to which such treatment influences pregnancy outcomes is not certain and is yet to be evaluated. Hence, this review intends to determine the influence of threatened miscarriage (TM) on maternal and perinatal outcomes and spontaneous abortion rate and also evaluate the various treatment protocols and their effects on fetal survival with the aim of suggesting an evidence-based approach to the care of such patients.

## Materials and methods

This was a retrospective cohort (case-control) study of women who met the diagnoses of threatened miscarriage and were managed in the maternity unit of the University of Calabar Teaching Hospital (UCTH), Calabar, Nigeria, between January 1, 2010, and December 31, 2019. The UCTH is a second-generation Nigerian tertiary health institution situated in the south-south region of the country.

Using the patient records and register in the maternity unit of the hospital, all cases of threatened abortions were identified and the case notes retrieved and analyzed. An equal number of asymptomatic pregnant women of similar age and parity with the same gestational ages who received antenatal care at the same period were systematically selected as control from the antenatal records. The diagnosis of threatened miscarriage was made by clinical and sonographic findings. The diagnostic criteria were based on documented history of vaginal bleeding with closed cervix before the gestational age of 20 weeks and ultrasound documented evidence of fetal heart activity at the time of presentation in the hospital [7,10].

Women with the following medical and pregnancy complications were excluded. They include diabetes mellitus, chronic hypertension, chronic renal disease, cardiac disease in pregnancy, known thrombophilia, antiphospholipid antibody syndrome, prior diagnosis of cervical incompetence, ultrasound evidence of fetal congenital anomaly, uterine anomaly, and uterine pathology such as fibroids, prior myomectomy, or cervical amputations. The outcome measures were pregnancy complications such as spontaneous abortion, antepartum hemorrhage (placenta abruptio and placenta previa), preeclampsia/eclampsia, pregnancy-induced hypertension, preterm labor and preterm birth, preterm pre-labor rupture of membranes (PPROM), mode of delivery, retained placenta, postpartum hemorrhage (PPH), low birth weight (<2.5Kg), birth asphyxia, neonatal ICU (NICU) admission, perinatal death, and neonatal sepsis.

Data were analyzed using IBM SPSS Statistics for Windows, Version 24.0 (Released 2016; IBM Corp., Armonk, New York, United States). Descriptive data were presented as frequencies and proportions. Logistic regression was computed to assess the relationship between dependent variables (pregnancy complications) and sub-groups (Independent variables). The statistically significant differences in mean BMI and the mean infant birth weights in the two groups were accessed using Z-test. The level of statistical significance was set at P < 0.05.

Formal approval was obtained from the UCTH Research Ethics Committee (protocol number UCTH/HREC/33/90) before the commencement of the study. Information obtained from patients' records was handled confidentially.

## Results

From available records, a total of 117 cases managed within the period under review met the diagnostic/inclusion criteria and were included in the analysis. An equal number of matched controls was also included in the final analysis. The mean BMI of the women was similar: 28.160 ± 5.082kg/m2 for the TM arm and 27.942± 4.418kg/m2 for the control arm (P = 0.798). Table 1 shows the demographic profile of the two groups of women. Among the women who experienced TM, 16 (13.7%), lost the pregnancy before fetal viability (spontaneous miscarriage) while 4 (3.4%) among the control had miscarriages. The difference was statistically significant (P = 0.005, OR: 4.475; 95% CI: 1.445 - 13.827). Among the women whose pregnancies progressed to fetal viability, there was a higher incidence of preterm delivery (before 37 weeks of gestation) among the TM group compared to the control, 10.9% versus 4.4%, (OR: 2.5; 95% CI: 1.75 - 3.65). In considering the mode of delivery, the study found that TM was associated with higher odds for cesarean delivery compared to the control (42.6% versus 25.7 %, OR: 1.7; 95% CI: 1.05 -2.96, P=0.02). Table 2 compares the incidences of adverse pregnancy outcomes. Women with TM had a statistically significantly higher incidence of placenta previa compared with the control (7.9% versus 1.8%; OR: 4.77; 95% CI: 2.19 - 23.04; P = 0.049). There were also statistically significantly increased odds for PPH and premature rupture of membranes (PROM). There was also no statistical difference in their mean birth weights: 3.113 ± 0.585kg for the TM group and 3.285 ± 0.536kg for the control (P=0.074). Table 3 summarizes other perinatal outcomes. In terms of intervention to improve pregnancy outcomes, the study revealed that admission to ward for in-patient care was a departmental policy routinely practiced. However, 11 of the women with TM declined admission and were managed as outpatient care. The main reason for in-patient care was bed rest. Analysis showed that five out of thirteen (36.5%) of the women who were managed on an outpatient basis miscarried their pregnancy compared to 11 out of 104 (10.6%) who had bed rest in the hospital until the bleeding stopped. (OR: 3.443, 95% CI: 1.70 - 7.99). Apart from bed rest, other interventions recorded include the administration of tocolytic drugs such as beta-agonists, antibiotics, progesterone supplements, sedatives, and cervical cerclage placement. The details of the interventions are shown in Table 4.

**Table 1:** Demographic and basic obstetrics profile TM - Threatened miscarriage

Variable		TM group (n=117)	Control (n=117)	P-value
Age Range	< 20 yrs.	6 (5.13%)	4 (3.42%)	P = 0.078
	20-29 yrs.	47 (40.17%)	41 (35.04%)	
	30-39 yrs.	61 (52.14%)	67 (57.27%)	
	≥ 40 yrs.	3 (2.56%)	5 (4.27%)	
Parity	Nulliparity	34 (29.06%)	42 (35.90%)	P=0.135
	Primiparity	40 (34.19%)	45 (38.46%)	
	Multiparity	43 (36.75%)	30 (25.64%)	
BMI (kg/m^2^)	<25	30 (25.64%)	25 (21.37%)	P=0.539
	25-29.9	53 (35.30%)	57 (48.72%)	
	30-34.9	24 (20.51%)	26 (22.22%)	
	≥35	10 (8.55%)	9 (7.69%)	

**Table 2:** Pregnancy complications PPH - Postpartum hemorrhage, SVD - Spontaneous vaginal delivery, C/S - Cesarean section, PROM - Premature rupture of membranes

Complications		Study cases (n=101)	Control (n=113)	P-value	Odds Ratio	95% Confidence Interval	
						Lower	Upper
Placenta previa	Yes	8 (7.9%)	2 (1.8%)	0.049	4.77	2.19	23.04
	No	93 (92.1%)	111 (98.2%)				
PPH	Yes	9 (8.9%)	4 (3.5%)	0.001	2.66	0.80	8.95
	No	92 (91.1%)	109 (96.5%)				
Mode of delivery	SVD	59 (58.4%)	84 (74.3%)	0.02	1.7	1.053	1.964
	C/S	42 (41.6%)	29 (25.7%)				
PROM	Yes	7 (6.9%)	1 (0.9%)	0.028	1.918	1.419	2.592
	No	94 (93.1%)	112 (99.1%)				
Placenta abruptio	Yes	2 (1.98%)	2 (1.8%)	1.000	1.051	0.394	2.816
	No	99 (98.02%)	111 (98.2%)				
Preeclampsia/eclampsia	Yes	5 (4.95%)	6 (5.3%)	0.905	0.851	0.495	1.806
	No	96 (95.05%)	107 (94.7%)				
Retained placenta	Yes	3 (2.97%)	2 (1.8%)	0.669	1.280	0.617	2.625
	No	98 (97.01%)	111 (98.2%)				

**Table 3:** Perinatal outcomes OR - Odds ratio, CI - Confidence interval, BW - Birth weight, LBW - Low birth weight, NICU- Neonatal ICU, SBA - Severe birth asphyxia

Perinatal Outcome			Study cases (n=101)	Control (n=113)	P-value	OR	95% C I	
							Lower	Upper
BW	LBW		10 (9.9%)	7 (6.2%)	0.317	1.273	0.832	1.948
	Normal		91 (90.1%)	106 (93.8%)				
NICU	Admitted		5 (4.95%)	2 (1.77%)	0.258	1.540	0.943	2.315
	Not Admitted		97 (95.05%)	111 (98.23%)				
Still Birth	Yes		2 (1.98%)	3 (2.66%)	0.553	0.844	0.386	2.494
	No		99 (98.02%)	110 (97.34%)				
SBA	Yes		2 (1.98%)	3 (2.66%)	0.686	0.700	0.224	2.101
	No		99 (98.02%)	110 (97.34%)				
Sepsis	Yes		4 (3.96%)	1 (0.89%)	0.191	1.724	1.086	2.736
	No		97 (96.04%)	112 (99.11%)				

**Table 4:** Effects of treatment/interventions on pregnancy outcome OR - Odds ratio, CI - Confidence interval

Intervention		Miscarriage n=16	Live birth n=101	OR	95% CI	
					Upper	Lower
Bed rest	Yes (n=104)	11 (10.6%)	93 (89.4%)	3.443	1.701	7.99
	No (n=13)	5 (36.5%)	8 (63.5%)			
Antibiotics	Treated (n=60)	8 (13.3%)	52 (86.7%)	1.008	0.873	1.165
	Not treated (n=57)	8 (14.0%)	49 (86.0%)			
Tocolytics	Treated (n=7)	2 (28.6%)	5 (71.4%)	0.818	0.510	1.315
	Not treated (n=110)	14 (12.7%)	96 (87.3%)			
Progestins	Treated (n=25)	4 (16%)	21 (84%)	0.966	0.80	1.167
	Not treated (n=92)	12 (13.0%)	80 (87%)			
Cerclage	Inserted (n=6)	2 (33.3%)	4 (66.7%)	0.924	0.592	1.440
	Not inserted (n=111)	14 (12.6%)	97 (87.4%)			

## Discussion

In this review, we found that vaginal bleeding in early pregnancy was a common pregnancy complication and the most common indication for admission during the first and second trimesters of pregnancy in this center. However, we included only cases that met the diagnostic criteria in the final analysis. Women who were managed and discharged without ultrasound evidence of fetal cardiac activity at the time of the bleeding event were excluded, which explains the relatively few cases analyzed within the period under review. The incidence of spontaneous miscarriage of 13.7% among women with TM compared to 3.4% in asymptomatic controls in this study indicates that TM is a warning sign for possible unwanted occurrence. This rate among TM pregnancies is similar to findings from other studies [10-12]. Threatened miscarriage is therefore a major risk factor for pregnancy loss before fetal viability.

Apart from the increased risk of spontaneous miscarriage, TM was associated with relatively increased odds of placenta previa, PROM and preterm birth compared with the asymptomatic women. This is in keeping with other studies [13-15]. Clinically, low lying placenta often presents as a warning bleed. Ultrasound imaging should be considered to ascertain placental location in women with a history of TM in the index pregnancy. Placenta previa is a major risk factor for PPH [16]. The incidence of other pregnancy complications such as preeclampsia and placenta abruption was similar in the two groups. 

The pathogenesis of TM and the adverse effects on pregnancy outcomes are not well understood. Abnormal placentation and implantation may result in first-trimester bleeding which if not resolved can progress to spontaneous abortion [17]. Molecular study has shown a significant increase in placental markers of oxidative stress in TM pregnancies [18]. Mal-expression of placental antioxidant enzymes and disruption in the balance of production of reactive oxygen radicals and the natural antioxidant defenses as well as endothelial damage leading to thrombi formation may negatively affect placental development and pregnancy outcome [19], hence the higher incidences of pregnancy complications. 

The increased cesarean delivery rate among patients with TM may have been affected by the higher incidences of placenta praevia and preterm birth, which are common indications for cesarean section. There were no significant differences in perinatal complication rates in the two groups of women. This is in keeping with other reports [20,21].

The treatment plans for women with TM were evaluated and the possible impact on their pregnancy outcomes assessed. The major interventions identified were bed rest, prophylactic antibiotics, progesterone therapy, cervical cerclage insertion, and administration of tocolytic drugs. However, our analysis suggests that only bed rest was effective in preventing pregnancy loss among women with TM. In a systematic review, prophylactic antibiotics did not reduce the risk of preterm rupture of membranes or preterm labor [22]. The effect of antibiotics was only observed in patients who had evidence of vaginal bacterial infection. Also, there was no difference in birth weight or neonatal sepsis similar to our findings [22].

Also, the use of tocolytics like magnesium sulfate and beta-2 adrenergic receptor agonists in women with TM in this study did not significantly prevent pregnancy loss. In some other studies, while magnesium treatment in women with low serum magnesium levels with preterm uterine contractions was beneficial, its use in patients with threatened miscarriage with absent uterine contractions did not improve pregnancy outcomes [23,24]. Furthermore, the success of emergency cervical cerclage insertion in preventing pregnancy loss in patients with TM is controversial. When prophylactic cerclage placement is offered to patients with ultrasound evidence of short or insufficient cervix, pregnancy outcome may improve, but its use in women with TM confers no significant benefit [25,26]. Similarly, there have been conflicting reports on the effectiveness of progesterone in the treatment of TM. A recent Cochrane review concluded that the available evidence suggests that progestogens probably make little or no difference in the live birth rate in women with TM [27].

Study limitations

The main limitation of this study is its retrospective nature. Sometimes vital information is missed in documentation; hence, the analysis was limited to cases that met diagnostic study criteria and adequate information concerning diagnosis, treatment, and outcome. Case notes with improper documentation were excluded from the final analysis.

A set of cofounders was controlled using regression analysis and the independent determinants/factors are presented. Wherever cofounders were encountered, path analysis was done to rule out the effect.

## Conclusions

At present, it is difficult to accurately predict which pregnancy with early pregnancy bleeding will eventually lead to miscarriage. The inability to accurately predict which pregnancy with threatened miscarriage will survive or end in spontaneous miscarriage may lead to unnecessary and potentially harmful or wasteful intervention. We recommend that women with TM should be offered bed rest in the hospital or if declined they should be counseled on modified bed rest at home. Psychological counseling and fetal surveillance should be offered to improve outcomes. The high probability of operative delivery should be discussed with the patients.
